# Do pharmacy practice standards effectively describe behaviour? Reviewing practice standards using a behavioural specificity framework

**DOI:** 10.1186/s12913-021-07358-4

**Published:** 2022-01-14

**Authors:** Deanna Mill, Amy Page, Jacinta Johnson, Kenneth Lee, Sandra M. Salter, Liza Seubert, Rhonda Clifford, Danielle D’Lima

**Affiliations:** 1 School of Allied Health, The University of Western Australia, Perth, Western Australia Australia; 2 Pharmacy Department, The Alfred, Melbourne, Victoria Australia; 3 Centre for Medicines Use and Safety, Monash University, Melbourne, Victoria Australia; 4 UniSA Clinical and Health Sciences, University of South Australia, Adelaide, South Australia Australia; 5 SA Pharmacy, SA Health, Adelaide, South Australia Australia; 6 Centre for Behaviour Change, Department of Clinical, Educational and Health Psychology, University College London, London, UK

**Keywords:** Pharmacist, Professional behaviour, Practice standards, Behavioural specification

## Abstract

**Background:**

Guidelines and practice standards exist to communicate the conduct and behaviour expected of health care professionals and ensure consistent quality practice. It is important that they describe behaviours explicitly so they can be interpreted, enacted and measured with ease. The AACTT framework specifies behaviour in terms of the: Action to be performed, Actor who performs the action, Context where the action occurs, Target who the action is performed with/for and Time when the action is performed (AACTT). It provides the most up to date framework for specifying behaviours and is particularly relevant to complex behavioural problems that involve sequences of behaviours performed by different people. Behavioural specificity within pharmacy practice standards has not been explored.

**Aim:**

To determine if behaviours described in the Professional Practice Standards for Australian Pharmacists specify Action, Actor, Context, Target and Time.

**Methods:**

Two researchers independently reviewed the scope and structure of the practice standards and one extracted action statements (behaviours) verbatim. Through an iterative process, the researchers modified and developed the existing AACTT definitions to operationalise them for application to review of the action statements in the practice standards. The operational definitions, decision criteria and curated examples were combined in a codebook. The definitions were consistently applied through a directed content analysis approach to evaluate all extracted action statements by one researcher. For consistency 20% was independently checked for agreement by a second researcher.

**Results:**

A novel codebook to apply AACTT criteria to evaluate practice standards was developed. Application of this codebook identified 768 independent behaviours. Of these, 300 (39%) described at least one discrete observable action, none specified an actor, 25 (3%) specified context, 131 (17%) specified target and 88 (11%) specified time.

**Conclusion(s):**

The behaviours detailed in practice standards for Australian pharmacists do not consistently specify behaviours in terms of Action, Actor, Context, Target and Time. Developers in the pharmacy profession, and beyond, should consider the behavioural specificity of their documents to improve interpretability, usability and adherence to the behaviours detailed. This also has implications for the development and evaluation of interventions to change such behaviours and improve quality of care.

Contributions to the literature

- Behavioural specificity within pharmacy professional practice standards has not been previously explored.
- Behaviours in practice standards need to be specified appropriately to ensure that 1) the user can read, understand and action them and 2) it is possible to observe, measure and assess influences on, the said behaviours to improve quality of care.
- The researchers applied the Actor, Action, Context, Target, Time (AACTT) behavioural specification framework to review, for the first time, the specificity of behaviours in the Australian professional practice standards for pharmacists and found that the behaviours are poorly specified.
- Thus, these standards cannot be used in their current form to assess and review the behaviour of pharmacists for professional development or to support behaviour change intervention development.

## Background

Practice standards are key to all aspects of healthcare [1]. Standards exist to promote delivery of consistent, high quality services and healthcare for patients, regardless of where or how they access the healthcare professional [2, 3]. They are often written by governing bodies and professional organisations to dictate expected behaviours (or actions) of individuals acting in a particular context [1, 4–7]. Practice standards vary in terms of their specific purpose and form however they usually detail the minimum expected behaviours and conduct of the professional (e.g. how a health care professional responds to a request for medical information) [6, 8]. Behaviours described in this context are perhaps better understood as actions, that is the things that the professional should do. For example, in the case of pharmacists, what they should do when dispensing a medication or providing medicines advice to a patient.

In Australia, the Professional Practice Standards for Pharmacists serve as a benchmark for pharmacists to ensure that they meet the expectations of the profession, society, other health professionals, funders and regulators [6]. To supply medicines subsidised by the government, Australian pharmacists must comply with these standards [6]. If pharmacists do not meet society’s, other health professionals’ or funding agencies’ expectations of them, the consequences can be devastating, for the community, the pharmacist and the pharmacy profession. As inadequate professional behaviours may lead to detrimental health outcomes for individuals or limits on the individual pharmacists professional practice [9, 10]. In such cases, practice standards can and have served as a framework to investigate alleged misconduct [6, 9]. If practice standards are ambiguous, they may be interpreted differently by different professionals and patients may not receive the expected level of patient centred care. Practising according to the standards is intended to ensure that legal, ethical and effective healthcare services are consistently provided by pharmacists regardless of practice setting.

Despite the central role practice standards play in communicating professional behaviours, useability of these documents has not been previously investigated. Clinical Guidelines serve a similar purpose to practice standards and usually communicate behaviours in terms of the considerations and decisions a clinician should make when providing care to a patient [2, 3] (e.g. what blood pressure management medication they should prescribe someone diagnosed with moderate hypertension). Broader research on clinical guidelines frequently cites clarity of content and language as essential in producing actionable guidelines [11–18]. In this body of research it is commonly stated that behaviours in such guidelines should be specific and unambiguous [11–19]. Concrete language and minimal ambiguity are likely essential to any document that communicates behaviours to its reader, including professional practice standards. Understanding how behaviours are expressed in practice standards would contribute to understanding the extent to which these documents are fit for purpose in communicating professional behaviour.

Michie and Johnston [16] have suggested that specifying who, what, when, where and how often a behaviour should occur in clinical guidelines would ensure a clear understanding of the behaviour and increase the likelihood of it being enacted [16, 19]. This level of specification also facilitates identification of barriers and enablers to the target population performing the behaviour [16, 19–21]. A rich understanding of the barriers and enablers to a target behaviour for a specific target population in a particular context can support the selection of theoretically congruent intervention components [16, 20, 21]. Such specification also supports the measurement of behaviours as part of intervention evaluation [18, 20–22]. Therefore, for the required behaviours to be measured, understood and improved, it is essential that practice standards are appropriately specified. This is vital for individuals to assess their own practice, and also for professional bodies or researchers to evaluate consistency and quality of practice within the profession.

Behaviour change science offers many theories, models and frameworks that support intervention development and evaluation. One such framework, The Behaviour Change Wheel [20, 21], outlines a variety of steps for intervention development starting with selection and specification of a target behaviour. The approach recommends that target behaviours are specified in terms of Who, What, When, Where, How Often and With Whom? [20, 21] Several other frameworks have been developed which are synonymous with and build on Michie’s ideas. These frameworks also focus on specifying behaviours as part of a behavioural intervention design process [22–25]. The first of these frameworks was the Target, Action, Context, Time (TACT) framework [24]. Next came its extension the Target, Action, Context, Timing and Actor (TACT-A) framework. Here it was recognised that the original TACT framework was limited as it assumed that an individual was performing the behaviour for themselves and did not specify ‘who’ needed to complete the action. This limited the practical application of the framework to behaviours where another individual is responsible for completing the action (e.g. a nurse taking a blood pressure reading for a patient), thus this extension included the addition of the ‘A: Actor – person who performs the action.’ [22, 25, 26] The most recent iteration of these frameworks is the Action, Actor, Context, Target, Time (AACTT) framework, that includes the Actor extension, additional detail added to the Target definition and a re-ordering of the components to improve its useability [23]. As well as being the most recent attempt to bring together the key criteria required for specifying behaviours, the AACTT framework is particularly useful when exploring complex behavioural problems that involve sequences of behaviours performed by different people [23]. For example, supplying a prescription medication to a patient would involve the prescriber, patient and a pharmacist, at a minimum. The authors wrote AACTT with the intention of specifying behaviour as a target for change, however they also suggested that ‘AACTT is compatible with any theory, model or framework in which behaviour is the focus of inquiry.’ [23] Thus, it is not unreasonable to think that this framework and the theory it draws upon may be useful in other contexts, albeit some adaptation to domains may be needed.

Previous work has adapted and applied the TACT-A framework for other purposes including, to analyse the specificity of behaviours in various document types [18, 26]. Gould et al. [26] utilised the TACT-A framework to review the content of feedback documents used to audit blood transfusion practice in healthcare settings in the United Kingdom. The results were intended to identify areas for improvement in these feedback processes and develop intervention guidance documents and training materials to improve these audit/feedback processes [26]. Similarly, in the United Kingdom, Smith and colleagues used the TACT-A framework to evaluate behaviours detailed in a policy documents from acute National Health Service trusts, to operationalise clinician responses to deteriorating patients in hospital wards [18]. They found a lack of specification of the Actor, Target, Time and Context that may be problematic in this setting and have since sought to further explore these actions and their influences, with the intention of designing a behavioural intervention to improve them [18, 27, 28]. These studies confirm that it is possible to adapt and use behavioural specification frameworks in more innovative ways to retrospectively assess specificity in different types of documents.

A literature review did not identify any previous research applying the AACTT framework to the review of behaviours in documents. Furthermore, no studies were found to review the useability of professional practice standards of any kind, let alone the specificity of the behaviours detailed in professional practice standards for pharmacists. As part of a broader project seeking to measure and influence the professional behaviour of pharmacists in Australia, the present study aimed to determine if behaviours described in the Professional Practice Standards for Australian Pharmacists specify Action, Actor, Context, Target and Time.

## Methods

### Professional practice standards for Australian pharmacists

**Fig. 1:**
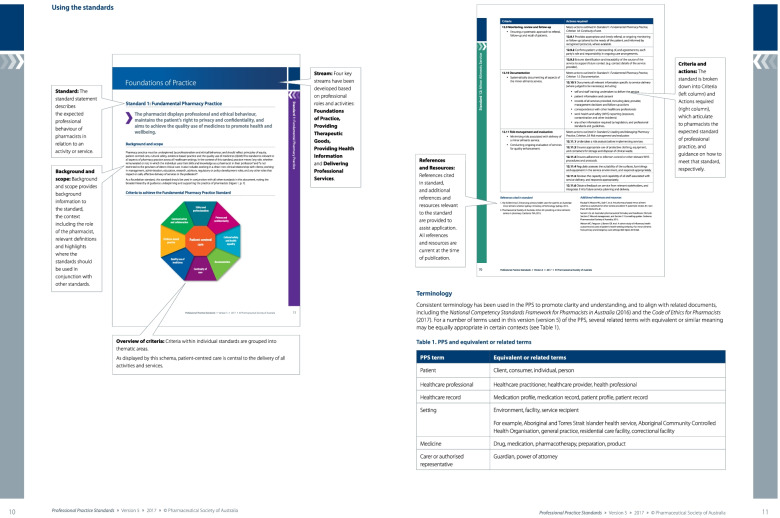
The layout of each standard and its components for the Pharmaceutical Society of Australia Professional Practice Standards [6]. (Authors have permission to reproduce this image from the Pharmaceutical Society of Australia)

This research reviewed the Professional Practice Standards for Australian Pharmacists, hereafter referred to as the practice standards [6]. This document applies to all practising pharmacists in Australia, regardless of role, scope, experience level or location of practice. Further, these practice standards have been reviewed and endorsed by all the relevant professional bodies, including the regulator, the Pharmacy Board of Australia. The practice standards contain 16 standards with independent statements entitled ‘actions required’ (referred to as action statements). For the 16 standards, each includes a preamble that consists of a standard statement along with the scope and background. Each standard lists the specific criteria and related actions that a pharmacist is required to perform to meet the standard. The action statements are presented as tabulated information alongside the standard criteria [6]. The document is intended to be read and interpreted in its entirety to provide context to each individual criteria and action statement (Fig. 1).

Given the practice standards are a text rich document a directed content analysis approach was undertaken [18, 26, 29, 30]. This type of analysis involves deductive coding of the text data where initial codes are derived from existing theory [29]. In this case, the theory that behavioural specification is important in documents that direct a professional’s behaviour and the AACTT behavioural specification framework criterion [16, 23]. The initial codes derived from the AACTT framework (which represents the existing theory) were then adapted through the development of operational definitions, before being systematically applied to code the text data for analysis with the intention of identifying themes and patterns. No specific guidelines for reporting content analysis were identified by the researchers. Given content analysis is traditionally a qualitative method this study is reported according to the Consolidated criteria for reporting qualitative studies (COREQ): 32-item checklist [31] (Appendix 1).

### Familiarisation and identification of behaviours

The practice standards were independently read and reviewed by two researchers (DM, DD). One researcher had a professional background as a pharmacist (DM) and the second as a behavioural scientist (DD), ensuring both relevant areas of expertise were represented in each stage of the study. The researchers focused attention on the structure and layout of the document with the aim to understand the location, sequence, presentation of information and the language use. During subsequent readings of the document, the researchers aimed to familiarise themselves with the presence of behaviours within the document. Behaviours were defined as actions that could or should be undertaken during the course of professional responsibilities, duties or employment as a pharmacist. Both reviewers independently determined that the action statement sections of each standard were the part of the document where behaviours were consistently described. Both researchers (DM, DD) agreed that the criteria, scope, background and standard statement sections did not contain specific actions but provided broad contextual information (e.g. Actor, Context, Target and Time) for the action statements.

### Data extraction

The first 12 pages of the practice standards and appendices were excluded from the analysis as these sections were considered to introduce, orientate and supplement the practice standards rather than provide actionable content. Therefore, data (action statements and accompanying information) were extracted from within the main body of the practice standards document from page 13 to 95.

One researcher (DM) extracted verbatim the action statements from the document and sought contextual information (Actor, Context, Target and Time) from preceding sections (criteria, scope, background and standard statement sections) if required. The actions were extracted as they contained the described behaviours. The extracted information included sufficient referencing to locate the information within the document (e.g. standard 2, criteria 3, action 5 was referred to as 2.3.5). Actions were excluded where they referred directly to other action statements (e.g. see “Meets actions outlined in Standard 1: Fundamental Pharmacy Practice, Criterion 1.1: Patient-centred care”) [6].

Details on the authors, version number, year published, publisher, scope and purpose of the practice standards were extracted for referencing and context.

### Deductive codebook development

The research team (DM, DD) used an iterative process to create a deductive code book that could be used to review the identified behaviours (action statements) against the Action, Actor, Context, Target, Time (AACTT) criteria (Table 1). To ensure consistent coding the researchers adapted the original AACTT criteria definitions for application in this context (reviewing practice standards), rather than its original purpose (specifying behaviours for behaviour change intervention design). Changes included revising the phrase ‘behaviour that needs to change’ to ‘behaviour’ for the definition of Action and clarifying that the definition of Time for this study included when a situation was dependent on a set of circumstances (Table 1). The action statements in the practice standards were also noted to refer to other documents (e.g. legislation, guidelines) essential to complete said actions. To account for these, additional criteria were added to the assessment of Action for the review, including assessment of whether the action referred to another document and if any other document referred to was adequately named and accessible (Table 1). A set of decision criteria was also developed (e.g. yes/no/more information needed/not necessary). Their associated definitions and example applications can be viewed in the code book (Table 1).

**Table 1:** Codebook developed to apply AACTT framework to analysis of the practice standards

Criteria*	AACTT Criteria original definition [23]	AACTT Criteria adjusted definition	Allocated codes and definitions	Examples from Professional Practice Standards for Australian Pharmacists
**ACTION** Is an action specified per the review definition?	A discrete observable behaviour that needs to change, in terms that can be observed or measured.	A discrete observable behaviour…in terms that can be observed or measured. *(Assess with* Table 2*)*	**Discrete action:** A single self-contained action without consideration to whether there are ancillary actions that may precede or proceed on from that single self-contained action. A non-discrete action may be the result of a series of discrete actions (e.g. the dispensing process or the medicine reconciliation process) though were considered non-discrete if it is stated without further description of the process. The assessment of whether the action is discrete includes an assessment of whether the reader is provided with the full details of the action or expected to bring pre-existing knowledge to interpret the action in a consistent manner. **Observable action:** Specified as an externally visible manifestation and/or the direct outcome of the action would be a physical object. Non-observable actions are those where there is no external physical sign that an action is performed. Yes - There is an action(s) that is/are discrete and observable in the statement. No - There is no discrete and observable action(s) present in the statement.	*Example Yes: ‘Obtains informed consent before delivery of services’* *Example No: ‘Supports principles of equity in the delivery of healthcare services’*
ACTION* **Reference to another document or guideline** Is there reference to another guideline or document in the action statement?		*Additional criterion added by research team.*	Yes - One or more guidelines or documents are mentioned in the statement. No - No guidelines or documents are mentioned in the statement.	*Example Yes: Understands and upholds relevant codes of ethics and codes of conduct.* *Example No: ‘Provides a setting for information exchange and service delivery that is appropriate to the patient.’*
ACTION* **Referenced guideline named and accessible** If there is reference to another guideline or document. Is it clear what the document/guideline is and how it can be accessed?		*Additional criterion added by research team.*	Yes - Guideline or document has been explicitly named and where it can be accessed in the statement. No -The guideline has not been explicitly named and where to access it has not been specified in the document. MIN -The guideline or document has been explicitly named OR how to access it is stated but not both.	*Example Yes: ‘Facilitates proactive referral and follow-up, as required. See Appendix 3: Template referral letter.’* *Example No: ‘Uses equipment for complex compounding that meets relevant Australian Standards’* *Example MIN: ‘This may include: preparation details (refer to ‘Extemporaneous dispensing’ in the current edition of Australian Pharmaceutical Formulary and Handbook)’*
**ACTOR** Is actor(s) specified per the review definition?	The individual or group of individuals who perform (or should/could) the Action.	The individual or group of individuals who perform (or should/could) the Action.	Yes -Explicitly names the person OR persons responsible for performing the action. No - No one is explicitly named to perform the action OR assumptions of responsible person need to made to interpret.	*Example Yes: ‘The accredited pharmacist …’* *Example No: ‘Self’ or ‘own’*
**CONTEXT** Is context specified per the review definition?	The physical location, emotional or social setting in which the Actor performs (or should/ could) the Action.	The physical location, emotional or social setting in which the Actor performs (or should/ could) the Action.	Yes - A location/context for the action has been explicitly named. No -A location/context has not been named OR assumptions need to be made to interpret the context. MIN -A location/context has been named but there could be multiple OR a reference to location/context is made but more information would be needed to interpret OR when nonspecific terms are used to refer to the context that could have multiple meanings and have not been predefined	*Example Yes: ‘Records in the dispensing history or patient healthcare plan and on the medicine label when initial brand substitution occurs.’* *Example No:’ Undertakes a risk analysis before implementing services.’* *Example MIN: ‘service environment’ or ‘workplace’*
**TARGET** Is target(s) specified per the review definition?	The individual or group of individuals for/ with/ on behalf of whom the Actor performs the Action.	The individual or group of individuals for/ with/ on behalf of whom the Actor performs the Action.	Yes -An individual or group that the action is with/for AND on behalf of is named. No - No individual or group that the action is with/for AND on behalf of is specified OR assumptions need to be made to interpret who the target is. MIN -When a reference to target is made but more information would be needed to interpret which specific individuals are the target OR one of with/for/behalf of is missing and shouldn’t be. NN - The action doesn’t require a target OR the target is also the actor.	*Example Yes: ‘with the patient’ or ‘discusses with the prescriber’* *Example No: ‘Communicates the evidence for the use of therapeutic goods clearly and transparently.’* *Example MIN: ‘Others’ or ‘staff’ or ‘team’ or ‘healthcare professionals’ (could be more specific eg. Pharmacy assistants or compounding technicians or prescriber, dentist, physiotherapist)* *Example NN: ‘Self-assess own knowledge and skills’*
**TIME** Is time specified per the review definition?	The time period and duration that the Actor performs the Action in the Context with/for the Target.	The time period and duration that the Actor performs the Action in the Context with/for the Target. *(For this study the research team will also consider where time is dependent on a set of circumstances if these have been explicitly stated.)*	Yes - Time period and duration if relevant and explicitly specified OR if states the situation when the action should occur. No -No time period or duration is specified, OR assumptions were made about them. MIN - When a reference to time is made but more information would be needed to interpret or assumptions would need to be made to interpret OR duration and/or frequency are present but not both.	*Example Yes: ‘Before supply of medication’ or ‘all mentoring opportunities’ or ‘when services are provided or refused’* *Example No: ‘Stores records safely, securely and in a dedicated location’* *Example MIN: ‘Timely’ or ‘regularly’ or ‘maintains’ or ‘as required’*

*MIN* More Information Needed

*NN* Not needed

*Additional criteria added by research team

The adapted AACTT criterion definitions were developed to assess the extracted data through the application of the associated decision criteria. Illustrative phrases were identified to support the decision-making. The application of the definition to the extracted action statements allowed the researchers (DM, DD) to assess if the statement met each AACTT criteria. If necessary, the researchers worked through the document in an iterative manner to identify if information pertaining to the four contextual criteria (Actor, Context, Target and Time) were available when the action statement was read in context (e.g. read with the previous sections of the standard, namely the criteria, scope, background and standard statement sections).

To ensure consistency, ambiguous verbs (used to describe the actions of a behaviour) identified in the practice standards were tabulated with their dictionary definition and whether their use indicated discrete and observable actions (Table 2). For example, the non-specific verb ‘ensure’ was identified in the document to describe behaviours (e.g. ensuring something is undertaken). Ensure was considered to be neither discrete nor observable so each time it occurred in the document it was coded as “No” (e.g. criteria not met). This table supported consistent analysis and application of the codebook during the coding process.

**Table 2:** Interpretation of action verbs as discrete and observable for the codebook

Action word	Relevant dictionary definitions from Macquarie Online Dictionary [32]	Interpretation of discrete and observable for review
**Ensure**	1. To secure, or bring surely, as to a person: *this letter will ensure you a hearing.* 2. To make sure or certain to come, occur, etc.: *measures to ensure the success of an undertaking.* 3. To make secure or safe, as from harm.	For all definitions: Not discrete as likely to take multiple successive actions. Not observable as not enough detail to know what the physical outcomes would be or what actions are needed.
**Maintain**	1. To keep in existence or continuance; preserve; retain: *to maintain good relations with New Zealand.* 2. To keep in due condition, operation, or force; keep unimpaired: *to maintain order*; *maintain public highways.* 3. To keep in a specified state, position, etc. 4. To affirm; assert (with a clause, or with an object and infinitive): *maintain that it is right*; *maintain it to be true.*	For all definitions: Not discrete and would take multiple successive actions. Some outcomes are possibly observable but adequate description of the action and outcome would be needed.
**Uses**	1. To employ for some purpose; put into service; turn to account: *use a knife to cut*; *use a new method.* 2. To avail oneself of; apply to one’s own purposes: *use the front room for a conference.* 3. To utter (words) or speak (a language). 4. To operate or put into effect.	For all definitions: Possibly discrete but dependent on if there is enough explanation as to ‘how’ to use the object, article etc. If ‘how’ is not clearly detailed then the action would be considered non-discrete. Some outcomes are possibly observable but adequate description of the action and outcome would be needed.
**Refer**	1. To direct the attention or thoughts of: *the asterisk refers the reader to a footnote.* 2. To direct for information or for anything required: *to refer students to books on a subject.* 3. (of a medical practitioner) to direct (a patient) to another doctor, usually a specialist, for further consultation or treatment. 4. To have recourse or resort; turn, as for aid or information: t*o refer to one’s notes.*	For definitions 1,2 and 4: Possibly discrete but dependent on if there is enough explanation as to ‘how’ and ‘what’ to refer the object, article etc. If ‘how’ and ‘what’ is not clearly detailed then the action would be considered non-discrete. Some outcomes are possibly observable but adequate description of the action and outcome would be needed. For definition 3: If referring to another health profession then this is likely discrete and observable.
**Identify**	1. To recognise or establish as being a particular person or thing; attest or prove to be as claimed or asserted: *to identify handwriting*; *to identify the bearer of a cheque.* 2. To serve as a means of identification for: *this card identifies the bearer as a member.*	For all definitions: Possibly discrete but dependent on if there is enough explanation as to ‘how’ and ‘what’ to refer the object, article etc. If ‘how’ and ‘what’ is not clearly detailed then the action would be considered non-discrete. Some outcomes are possibly observable but adequate description of the action and outcome would be needed.
**Liaise**	1. To maintain contact and act in concert.	Not discrete as likely to take multiple successive actions. To be discrete would need an explanation of ‘how’ this is being done. Possibly observable but this is dependent on specific outcome or action.
**Monitor**	1. To check, observe, or record the operation of (a machine, etc.), without interfering with the operation. 2. To supervise; observe critically.	For all definitions: Not discrete as likely to take multiple successive actions. To be discrete would need an explanation of ‘how’ this is being done. Possibly observable but this is dependent on specific outcome or action.
**Adhere**	1. To be devoted to; be attached to as a follower or upholder: *to adhere to a party*; *to adhere to a leader*; *to adhere to a church*; *to adhere to a creed.* 2. To hold closely or firmly to: *to adhere to a plan.*	For all definitions: Not discrete as likely to take multiple successive actions. To be discrete would need an explanation of ‘how’ this is being done and ‘what’ is being adhered to. Possibly observable but this is dependent on specific outcome or action.
**Implement**	1. To put (a plan, proposal, etc.) into effect	Not discrete as likely to take multiple successive actions. Not observable as not enough detail to know what the physical outcomes would be or what actions are needed.
**Consider**	1. To contemplate mentally; meditate or reflect on. 2. To regard as or deem to be: *I consider the examination is justified.* 3. To think; suppose. 4. To make allowance for. 5. To pay attention to; regard: *he never considers others.*to view attentively, or scrutinise.	For all definitions: Most likely not discrete and observable as is an internal thought process, may take multiple actions in succession to achieve and will not necessarily always have a physical outcome.
**Review**	1. To inspect, especially formally or officially. 2. To look back upon; view retrospectively.	For all definitions: Not discrete as likely to take multiple successive actions. To be discrete would need an explanation of ‘how’ this is being done and ‘what’ is being reviewed. Possibly observable but this is dependent on specific outcome or action.
**Appraise**	1. To estimate generally, as to quality, size, weight, etc. 2. To value in current money; estimate the value of.	For all definitions: Not discrete as likely to take multiple successive actions. To be discrete would need an explanation of ‘how’ this is being done and ‘what’ is being appraised. Possibly observable but this is dependent on specific outcome or action.
**Evaluate**	1. To ascertain the value or amount of; appraise carefully.	Not discrete as likely to take multiple successive actions. To be discrete would need an explanation of ‘how’ this is being done and ‘what’ is being evaluated. Possibly observable but this is dependent on specific outcome or action.
**Assess**	1. To fix or determine the amount of (damages, a tax, a fine, etc.). 2. To measure or evaluate.	For all definitions: Not discrete as likely to take multiple successive actions. To be discrete would need an explanation of ‘how’ this is being done and ‘what’ is being assessed. Possibly observable but this is dependent on specific outcome or action.
**Accesses**	1. To gain admittance to: *you can access the foyer through this door.*	Discrete and observable as is a physical action.
**Encourages**	1. To inspire with courage, spirit, or confidence. 2. To stimulate by assistance, approval, etc.	For all definitions: Not discrete as likely to take multiple successive actions. To be discrete would need an explanation of ‘how’ this is being done. Possibly observable but this is dependent on specific outcome or action.
**Facilitates**	1. To make easier or less difficult; help forward (an action, a process, etc.). 2. To assist the progress of (a person): *to facilitate the customer to find the right product.*	For all definitions: Not discrete as likely to take multiple successive actions. To be discrete would need an explanation of ‘how’ this is being done. Possibly observable but this is dependent on specific outcome or action.
**Engage**	1. To occupy the attention or efforts of (a person, etc.): *she engaged him in conversation.* 2. To secure for aid, employment, use, etc.; hire: *to engage a worker*; *to engage a room.* 3. To attract and hold fast: *to engage the attention*; *to engage someone’s interest.* 4. To reserve or secure. 5. To attract or please: *his good nature engages everybody he meets.* 6. To bind as by pledge, promise, contract, or oath; make liable. 7. To occupy oneself; become involved: *to engage in business*; *to engage in a strategy.*	For all definitions: Not discrete as likely to take multiple successive actions. To be discrete would need an explanation of ‘how’ this is being done. Possibly observable but this is dependent on specific outcome or action.
**Confirm**	1. To make certain or sure; corroborate; verify: *this confirmed my suspicions.* 2. To make valid or binding by some formal or legal act; sanction; ratify: *to confirm an agreement.* 3. To reaffirm (a booking, ticket reservation, appointment, etc.) as by a notification to a person or organisation of one’s intention to carry out one’s original plans. 4. To strengthen (a person) in habit, resolution, opinion, etc.	For definitions 2 and 3: Discrete and observable if ‘what’ or ‘who’ and ‘how’ something is being confirmed is named, as likely to have a physical or verbal outcome. For definitions 1 and 4: Not discrete as likely to take multiple successive actions. These may be internal thought processes. To be discrete would need an explanation of ‘how’ this is being done. Possibly observable but this is dependent on specific outcome or action.
**Selects**	1. To choose in preference to another or others; pick out.	Possibly discrete but may involve internal decision-making processes. Likely observable if physically choosing between objects.
**Recommends**	1. To commend by favourable representations; present as worthy of confidence, acceptance, use, etc.: *to recommend a book.* 2. To represent or urge as advisable or expedient: *to recommend caution.* 3. To advise (a person, etc., to do something): *I recommend you to wait.*	Discrete and observable as likely to have a physical or verbal outcome.

### Application of codebook

Once both researchers (DM, DD) were satisfied with the definitions, the standardised deductive codebook was applied to the extracted action statements using a directed content approach. One pharmacist researcher (DM) initially applied the codebook to the action statements. A second researcher (DD) independently checked the coding for 20% of the data pertaining to the individual action statements. The data (action statements) for the final check were selected through use of a random number generator. The row of data (action statement) from the database that corresponded to the number generated was then checked by the second researcher (DD). These included a selection of data (*n* = 154, 20% of action statements) from each of the 16 standards. Any differences identified during this check were resolved through discussion (DM, DD). The definitions were clarified again at this time to reduce ambiguity in interpretation by the researchers. One researcher (DM) then went through and reviewed all of the data to reflect the minor clarification changes that had been made to the codebook as a result of the 20% check.

### Data analysis

Results were summarised for each criterion and code using frequencies and proportions calculated in the latest version of Microsoft Excel.

## Results

The practice standards contain 768 independent action statements over the 16 different standards. The assessments for the Action, Actor, Context, Target and Time criteria of the action statements from ‘Standard 1- Fundamental Pharmacy Practice’ through to ‘Standard 16- Harm Minimisation’, are presented in Tables 3 and 4. The summary of the background, standard statements, and criteria assessments for Actor, Context, Target and Time can be viewed in Table 5. The application of the criteria and potential improvements have been presented using extracted action statements as illustrative examples in Table 6. Results for each component of the AACTT will be discussed separately below.

**Table 3:** Summary of all action statements ACTION criteria analysis by standard

	Number with behaviourally relevant content	Action statements with discrete observable actions	Action statements with more than one discrete observable actions	Action statements that’s ACTION referenced other documents	Action statements that’s reference to other documents explicitly named and said how to access the document	
Standard	No.	Meets criteria No. (%)	No. (%)	No. (%)	Specified No. (%)	Partially specified No. (%)
Standard 1: Fundamental Pharmacy Practice	48	22 (46%)	2 (4%)	8 (17%)	4 (50%)	0
Standard 2: Leading and Managing Pharmacy Practice	51	16 (31%)	1(2%)	11(22%)	0	1 (9%)
Standard 3: Dispensing and Other Supply Arrangements	70	36 (51%)	3 (4%)	14 (20%)	1 (7%)	4 (29%)
Standard 4: Provision of Non-prescription Medicines and Therapeutic Devices	30	16 (53%)	2 (7%)	7 (23%)	0	2 (29%)
Standard 5: Compounding	75	36 (48%)	10 (13%)	22 (29%)	6 (27%)	5 (23%)
Standard 6: Medicines Information	23	3 (13%)	1 (33%)	0	0	0
Standard 7: Health Promotion and Education	21	6 (29%)	0	1 (5%)	0	0
Standard 8: Counselling	36	14 (39%)	1 (3%)	4 (11%)	0	0
Standard 9: Collaborative Care	52	12 (23%)	1 (2%)	7 (13%)	0	0
Standard 10: Screening and Risk Assessment	36	10 (28%)	0	10 (28%)	2 (20%)	2 (20%)
Standard 11: Vaccination Service	65	26 (40%)	1 (2%)	14 (22%)	0	4 (29%)
Standard 12: Minor Ailments Service	39	13 (33%)	0	9 (23%)	1 (11%)	1 (11%)
Standard 13: Disease State Management	47	16 (34%)	1 (2%)	10 (21%)	2 (20%)	3 (30%)
Standard 14: Medication Review	52	31 (60%)	3 (6%)	10 (19%)	1 (10%)	0
Standard 15: Dose Administration Aid Service	73	21 (29%)	0	10 (14%)	2 (20%	1 (10%)
Standard 16: Harm Minimisation	50	22 (44%)	0	11 (22%)	1 (9%)	2 (18%)
**Total**	768	300 (39%)	26 (3%)	148 (19%)	20 (14%)^a^	25 (17%)^a^

% calculated per total no. of behaviourally relevant actions in each standard unless otherwise specified

^a^% calculated per total action statements that referenced other documents

**Table 4:** Summary of action statements ACTOR, CONTEXT, TARGET and TIME criteria analysis by standard

	Actions with behaviourally relevant content	ACTOR(s) was specified	CONTEXT was specified		TARGET(s) was specified			TIME was specified	
Standard	No.	Meets criteria No. (%)	Meets criteria No. (%)	Partially meets criteria No. (%)	Meets criteria No. (%)	Partially meets criteria No. (%)	Not necessary No. (%)	Meets criteria No. (%)	Partially meets criteria No. (%)
Standard 1: Fundamental Pharmacy Practice	48	0	0	5 (10%)	14 (29%)	23 (48%)	1 (2%)	8 (17%)	6 (13%)
Standard 2: Leading and Managing Pharmacy Practice	51	0	0	6 (12%)	2 (4%)	28 (55%)	7 (14%)	2 (4%)	7 (14%)
Standard 3: Dispensing and Other Supply Arrangements	70	0	5 (7%)	2 (3%)	17 (24%)	28 (40%)	2 (3%)	12 (17%)	9 (13%)
Standard 4: Provision of Non-prescription Medicines and Therapeutic Devices	30	0	1 (3%)	0	8 (27%)	11 (37%)	2 (7%)	7 (23%)	4 (13%)
Standard 5: Compounding	75	0	3 (4%)	8 (11%)	4 (5%)	14 (19%)	5 (7%)	14 (19%)	20 (27%)
Standard 6: Medicines Information	23	0	0	1 (4%)	6 (26%)	13 (57%)	1 (4%)	2 (9%)	3 (13%)
Standard 7: Health Promotion and Education	21	0	1 (5%)	0	1 (5%)	14 (67%)	1 (5%)	2 (10%)	1 (5%)
Standard 8: Counselling	36	0	2 (6%)	0	11 (31%)	10 (28%)	0	3 (8%)	3 (8%)
Standard 9: Collaborative Care	52	0	0	2 (4%)	2 (4%)	31(60%)	2 (4%)	1 (2%)	4 (8%)
Standard 10: Screening and Risk Assessment	36	0	1 (3%)	0	7 (19%)	8 (22%)	4 (11%)	3 (8%)	7 (19%)
Standard 11: Vaccination Service	65	0	0	4 (6%)	12 (18%)	29 (45%)	2 (3%)	8 (12%)	12 (18%)
Standard 12: Minor Ailments Service	39	0	0	2 (5%)	6 (15%)	13 (33%)	0	3 (8%)	6 (15%)
Standard 13: Disease State Management	47	0	0	1 (2%)	10 (21%)	19 (40%)	4 (9%)	2 (4%)	5 (11%)
Standard 14: Medication Review	52	0	1 (2%)	3 (6%)	11 (21%)	25 (48%)	0	4 (8%)	7 (13%)
Standard 15: Dose Administration Aid Service	73	0	8 (11%)	0	10 (14%)	22 (30%)	3 (4%)	12 (16%)	16 (22%)
Standard 16: Harm Minimisation	50	0	3 (6%)	1 (2%)	10 (20%)	15 (30%)	2 (4%)	6 (12%)	9 (18%)
**Total**	768	0	25 (3%)	35 (5%)	131(17%)	303 (39%)	36 (5%)	88 (11%)	120 (16%)

% calculated per total no. of behaviourally relevant actions in each standard

**Table 5:**
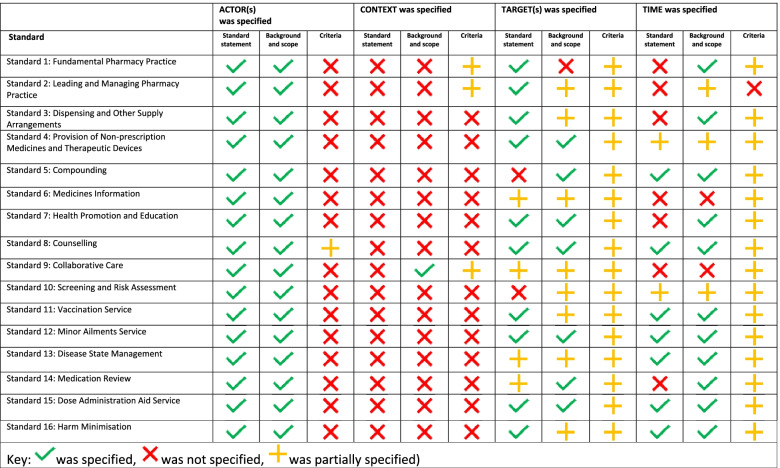
Summary of Standard statement, Background and scope, and Criteria ACTOR, CONTEXT, TARGET and TIME criteria analysis by standard

**Table 6:** Example action statements specification problems and possible adjustments

	Example number and problem type	Example action statement verbatim from the standards (4)(relevant section bolded)	Problem explained	Example of possible specification adjustment for the problem (relevant section bolded) ^**a**^	Other issues with this criteria	Example of possible specification for entire action statement per AACTT (adjusted sections bolded) ^**a**^
1.	Not a discrete or observable action	3.8.8 **Creates awareness** about the availability and appropriateness of brand substitution, and the contribution it makes to the sustainable use of health resources.	Creating awareness is difficult to observe and would likely require numerous discrete actions. This term needs to be replaced with something that is discrete and observable e.g. Educates.	**Educates on** the availability and appropriateness of brand substitution, and the contribution it makes to the sustainable use of health resources.	Actor, context, target and time are all not specified. Assume actor is pharmacist, patient is target, context is in the pharmacy and time could be when they are dispensing medicines for them.	**The pharmacist educates patients presenting to the pharmacy on the** availability and appropriateness of brand substitution, and the contribution it makes to the sustainable use of health resources **when the patient presents for a medication.**
2.	Not an observable action	3.8.7 **Ensures** that any substitution is **directly, clearly and appropriately discussed and/or demonstrated with the patien**t in the counselling process.	We can’t observe someone ensuring something. We can observe them discussing substitution with the patient. Suggest remove ‘ensures’ and focus on discussing as the action.	**Discusses and/or demonstrates** substitution directly, clearly and appropriately with the patient in the counselling process.	Actor and context are not specified, assume pharmacist and in the pharmacy (doesn’t necessarily have to be though). Time is during the counselling process but how often should this occur, assume always when substitution has been made. Should name what is being substituted e.g. medication.	**The dispensing pharmacist discusses and/or demonstrates medication** substitution directly, clearly and appropriately with the patient in the counselling process **whenever brand substitution is made.**
3.	How the action should be performed needs clarification	15.7.5 **Maintains** accurate and clear records of all medications packed into DAAs (e.g. date, quantity, instances where brand substitution occurred, packed by [signature], checked by [signature]).	What is required to maintain these clear records? Assume need to document each of those things into a record every time they are packed into a DAA for a patient.	**Documents (in an ongoing record)** all medications packed into DAAs (e.g. date, quantity, instances where brand substitution occurred, packed by [signature], checked by [signature]).	Actor is not specified, assume pharmacist. Target and time are not specified, assume patient and every time it is packed.	**The pharmacist documents (in an ongoing record)** all **medications packed into the DAA for the patient, every time (**e.g. date, quantity, instances where brand substitution occurred, packed by [signature], checked by [signature]).
4.	Nonspecific language used, how to perform action not specified	14.8.2 **Confirms** that the patient, or carer or authorised representative, understands the process, requirements, benefits and limitations of **the service**.	How should they confirm this, could specify verbally? The service could also be further specified, in this standard it is talking about medicine review services.	**Verbally confirms** that the patient, or carer or authorised representative, understands the process, requirements, benefits and limitations of **the medicines review service**.	Actor, context and time are missing. Assume accredited pharmacist is the actor, context would be specific to the type of medicines review and this should all be confirmed prior to initiating the service.	**The accredited pharmacist verbally confirms** that the patient, or carer or authorised representative, understands the process, requirements, benefits and limitations of **the medicines review service prior to conducting the medicine review**.
5.	Reference to document with necessary information missing	14.3.1 Ensures that all medication review services are consistent with **relevant clinical guidelines and comply with program guidelines**.	Reference to clinical and program guidelines, but not specified exactly what they are or where they can be found. Suggest naming the relevant documents and supplying a link to where they can be found.	Ensures that all medication review services are consistent with relevant clinical guidelines and comply with program guidelines **for medicine review services**. **These guidelines can be accessed at** www.findthedocumenthere.com	Ensures isn’t discrete or observable, suggest changing to provides all medication review services that are… Actor isn’t specified, should be an accredited pharmacist. Target isn’t specified but assume is a patient. Context isn’t specified but would be dependent on the type of service being provided.	**The accredited pharmacist always provides** medication review services **to patients** that are consistent with **relevant clinical guidelines and comply with program guidelines for medicine review services**. **These guidelines can be accessed at** www.findthedocumenthere.com
6.	Actor not specified	2.3.2 Works with a mentor for peer review of practice or to assist in meeting professional development goals.	Who works with a mentor? The actor is not specified.	**All pharmacists** should work with a mentor for peer review of practice or to assist in meeting professional development goals.	Action is ‘works’ and ‘peer review of practice’ could both be further specified. Context and Time are also not specified at all.	**All pharmacists should meet with a mentor in their own workplace, at least once a year so that the mento**r can peer review their daily practice or assist the pharmacist to meet their professional development goals.
7.	Assuming actor is a pharmacist but which pharmacist	11.3.5 Confirms that the authorised immuniser has professional indemnity insurance and that the delivery site has insurance policies appropriate for the delivery of a vaccination service.	Whose job is it to do this? Is it the pharmacy owner, the pharmacist manager, the pharmacist on duty or someone else?	**The pharmacy owner** confirms that the authorised immuniser has professional indemnity insurance and that the delivery site has insurance policies appropriate for the delivery of a vaccination service.	Time is not specified at all. Context is delivery site but could be further specified. Confirms could also be further specified- how should they confirm? For example, verbally, via citation of appropriate documentation.	**Before providing an immunisation service from the pharmacy the pharmacy owner confirms through citation of appropriate documentation** that the authorised immuniser has professional indemnity insurance and that the delivery site has insurance policies appropriate for the delivery of a vaccination service.
8.	No context specified but action may be in a fixed location	16.6.9 Adapts workflow to facilitate the delivery of harm minimisation services.	Workflow and the delivery of the harm minimisation service where?	Adapts workflow of **the dispensary and front of shop** to facilitate the delivery of harm minimisation services **in the community pharmacy**.	Adapting is not a discrete behaviour as it would require multiple actions to achieve or would depend on need. Target is not specified and, in this case, could be for the patient and with the pharmacy owner and staff. Time is not specified, but likely should be prior to providing the service. Actor is also not specified and could be the pharmacy owner, manager, and/or dispensing pharmacist.	**Prior to providing** harm minimisation services **to patients, the dispensing pharmacist and pharmacy assistants** adapts the workflow of **the dispensary and front of shop** to facilitate the delivery of harm minimisation services **in the community pharmacy**.
9.	Non-specific reference to a physical location/context	12.11.4 Regularly assesses the suitability of the surfaces, furnishings and equipment in the **service environment**, and responds appropriately.	Service environment is non-specific? Standard 12 refers to Disease State Management services that would like to occur in a community pharmacy and preferably in a private consultation room within that pharmacy.	Regularly assesses the suitability of the surfaces, furnishings and equipment in the **consultation room where disease state management services are provided** and responds appropriately. OR Regularly assesses the suitability of the surfaces, furnishings and equipment in the **consultation room of the pharmacy** and responds appropriately.	Assess and responds appropriately are not necessarily observable or discrete as actions. Actor is missing, assume it should be pharmacist. Target is not specified, assume this is done for the patient. Time is specified as, regularly but this is not specific and could be further specified eg. Weekly or prior to providing the service.	**Prior to providing a service the pharmacist** assesses the suitability of the surfaces, furnishings and equipment in the **consultation room where disease state management services are provided to patients…**
10.	Non-specific term used for the target	5.11.3 Educates other **health professionals** about appropriate preparation and administration of complex compounded preparations.	The target is health professionals, but this is a non-specific term and could be further specified as to who is most likely to need education on preparation and administration. This would most likely be nurses as they usually administer medications.	Educates **nurses responsible for administration** about appropriate preparation and administration of complex compounded preparations.	Actor is missing, assume pharmacists. Action is educating, could be further specified how they should educate. Context and time are also not specified, but context may not matter for education and time could be as requested or prior to them administering.	The pharmacist educates **nurses responsible for administration of medications** about appropriate preparation and administration of complex compounded preparations, **when requested by the nurses to do so**.
11.	Missing information for target	8.7.1 Checks the dispensing history and/or electronic healthcare record to determine the appropriateness of information being sought and provided.	Does not specify who is seeking the information and who it is being provided to. Assume target is a patient.	Checks the dispensing history and/or electronic healthcare record to determine the appropriateness of information being sought **by** and provided **to a patient.**	Actor is not specified, assume pharmacist. Action words are checks and determines, not necessarily observable and discrete. Context is not specified, assume in a pharmacy. Time is not specified assume, when it is requested.	**Upon request, the pharmacist will** check the dispensing history and/or electronic healthcare record **of a patient** presenting **in a pharmacy,** to determine the appropriateness of information being sought **by** and provided **to them.**
12.	Assumption of when to do action is needed	4.8.2 Provides advice to optimise use of non-prescription medicines and therapeutic devices.	It is not specified when this should be done. Assume this should be done every time either of these products is supplied or it is requested by the patient.	**When asked by the patient** provides advice to optimise use of non-prescription medicines and therapeutic devices. OR Provides advice to optimise use of non-prescription medicines and therapeutic devices **every time one of these products is supplied.**	Actor is not specified, assume pharmacist. Context is not specified, assume within the pharmacy or wherever selling/recommending these products. Target is not specified, assume a patient.	**When asked by a patient the pharmacist** provides advice to optimise use of non-prescription medicines and therapeutic devices **sold in the pharmacy or elsewhere**.
13.	Non-specific language used for time	5.5.6 **Regularly** conducts and documents environmental monitoring of the cleanroom and ancillary areas, and initiates corrective action, **as required**.	Nonspecific terms ‘regularly’ and ‘as required’ have been used to indicate when the action should be completed. These could be further specified by stating a length of time instead of regularly and a specified circumstance instead of as required.	Conducts and documents environmental monitoring of the cleanroom and ancillary areas **every day**, and initiates corrective action, **when results differ from normal range**.	Actor is not specified and, in this case, could be a pharmacist or assistant assigned by the pharmacist. Action words conducts, documents and initiates corrective action could possibly be further specified (e.g. how should it be conducted, what is corrective action). Context is specified as clean room and ancillary areas, but no context for where it should be documented has been provided. Target is not specified but this would depend if the pharmacist or an assistant was completing the action.	The **pharmacist** conducts and documents environmental monitoring of the cleanroom and ancillary areas **in the monitoring log every day**, and initiates corrective action, **when results differ from normal range**.

^a^Assumptions made to interpret/make example adjustment

### Is ACTION specified in the action statements of the practice standards?

At least one discrete and observable behaviour was described in over one-third of action statements (*n* = 300, 39%) (Table 3). A further 26 (3%) detailed more than one discrete and observable behaviour. Data for the assessments on actor, context, target and time criteria for each action statement is summarised in Table 4. Most times where the specificity criteria were not met, this was due to the verbs used to describe the action (Table 6, examples 1–4). One hundred and forty-eight (19%) action statements referenced other documents, legislation or guidelines necessary to access or understand in order to complete the specified action. Details to facilitate access to these additional resources were present in a minority of statements (*n* = 20, 14%). Partial resource details were missing for 25 (17%) of these additional resources (Table 6, example 5).

### Is ACTOR specified in the action statements of the practice standards?

None of the action statements specified an actor (Table 4). It is likely assumed that the individual reading the document would be a pharmacist and that the pharmacist is the actor in each action statement. However, sometimes further clarity about which pharmacist should complete the action would assist with interpretation (e.g. pharmacist accredited to provide immunisations or pharmacy owner) (Table 6, examples 6 and 7). While ‘pharmacist’ was not specified as the actor in the criteria of each standard, it was specified in the standard statement and background and scope sections (Table 5).

### Is CONTEXT specified in the action statements of the practice standards?

Twenty-five (3%) action statements explicitly specified a context for the physical location. A further 35 (5%) provided partial information regarding context (Table 4). Context was not specified in any standard statements. Context was specified once in the background and scope of one standard, and rarely in criteria of each standard (Table 5). There were several action statements that utilised the word ‘service’ with an inference to referencing a physical location. While a ‘service’ may have referred to a fixed physical location and therefore may have provided context, it could not be ascertained clearly that this was the case (Table 6, example 8). Some action statements had a broad reference to a physical location such as ‘workplace,’ however these could also be further specified or defined for each standard (Table 6, example 9).

The AACTT definition of context includes emotional context (e.g. stressful or calm) and social setting (e.g. one-on-one interaction or with both patient and carer present) in addition to the physical location. The researchers did not detect any action statements that provided either emotional context or social setting in the analysis.

### Is TARGET specified in the action statements of the practice standards?

The target was specified in 131 (17%) action statements and 36 (5%) did not require a target (Table 4). A further 303 (39%) provided partial information on the target. This information was specified or partially specified in the standard statement, background and scope and criteria of each standard (Table 5). Most times non-specific terms such as ‘staff’ or ‘other healthcare workers’ were used when further specification could have been made (Table 4, example 10). In other cases, the ‘on behalf of’ was specified, but the ‘who with’ required further clarification (Table 6, example 11).

### Is TIME specified in the action statements of the practice standards?

Time was specified within 88 (11%) action statements with partial information provided by a further 120 (16%) action statements (see Table 4). Time was at least partially specified in most standard statements, background and scope and criteria for each standard (Table 5). Action statements sometimes stated that an action should be completed ‘in a timely manner’ which refers to how it should be completed rather than when. In other action statements, it was stated that the action should be undertaken ‘as required,’ which requires the reader to interpret the information (Table 6, example 12). It was rarely specified how often the action should occur. While it may be intended that the reader should assume the actions are ongoing when providing a service, this inference is not explicitly stated (Table 6, example 13).

## Discussion

This study sought to determine if behaviours described in the Professional Practice Standards for Australian Pharmacists specify Action, Actor, Context, Target and Time. Results demonstrated that these behaviours are not consistently specified in this way. The practice standards poorly specified action, most commonly due to the verbs used in the action statements being non-discrete and/or non-observable. Actor, Context, Target and Time, or a combination of these, were frequently missing or described ambiguously in the standards. The standards do not provide descriptive behaviours and instead detail broad behaviours in a non-specific manner. In their current form the standards require significant interpretation by the reader and would not be useful as a tool to measure, observe or design interventions to influence the professions’ behaviour.

Smith et al. [18] found that the ‘action statements’ in policy documents intended to operationalise clinician responses to deteriorating patients in acute National Health Service hospital wards, more frequently specified all elements of the TACT-A criteria than the present study. ‘Action’ was the most frequently specified element in their analysis and in most cases they graded this as being reported specifically. Despite Smith et al. [18] more frequently identifying each criteria, similar issues were noted in this study for those criteria that were not specified adequately including: ‘Time’ was poorly specified owing to the use of terms that referenced a time but that still may be interpreted differently between individuals (e.g. urgently or timely) and ‘Actor’ was the least frequently specified element likely due to the documents assuming that the actor (or reader) should know who they are. Differences in results are likely due to the nature of the documents reviewed and the context in which they are used. The deteriorating patient documents reviewed by Smith et al. [18] likely provided more prescriptive actions given that they were policy documents and the behaviours detailed may be the difference between life and death for a hospitalised patient [18]. In comparison, the practice standards seem to be broad behavioural statements as they apply to an entire profession of individuals in all practice settings and roles.

Gupta et al. [13] explored sample recommendations from several Canadian clinical treatment guidelines (e.g. diabetes, asthma and ischaemic heart disease management) and discussed how they could be improved in terms of further specifying ambiguous terms. As in our study, the authors recognised that by not specifying exactly who (Actor) should do what, ‘task ambiguity’ is introduced [13]. Similarly, this study found that a lack of context introduces ‘semantic ambiguity,’ where when taken out of context, the word or phrase (in our case behaviour) could have more than one interpretation [13]. While our work focussed on practice standards, that may serve a different purpose to clinical guidelines, considerations for specifying behaviours and recommendations are indeed similar.

### Implications

The findings of this study demonstrate that, despite practice standards outlining the behaviours expected of the pharmacy profession, specificity has not been appropriately considered. While a high level of generalisability in these statements may be considered necessary when they are intended to apply to many different individuals in many different practice settings, the more generalisable they are the more difficult they are to use (i.e. for individuals to understand the minimum expected professional behaviours of pharmacists) [13, 19, 20, 25]. This also limits their applicability to measure and evaluate the practice of pharmacists, explore influences on the desired behaviours and develop interventions to increase their likelihood of occurrence.

The lack of specificity identified may lead to inconsistent interpretation of the behaviours contained and as a result inconsistent practice by Australian pharmacists. This may be particularly problematic for pharmacist-patient relationships that are built on trust, where inconsistency in interactions for medicines and medicines information between pharmacist may confuse or undermine this relationship, preventing provision of care [33, 34]. Where a pharmacist’s practice is under review by the Pharmacy Board of Australia or other regulator there may be a disconnect between the expectation and interpretation of the standards by the regulator versus the pharmacist, resulting in confusion and at worst, reprimand. Similar issues may arise where practising to the standards is required for renumeration, where the viability of the service may be put at risk due to unclear expectations. Further, if behaviours in the standards are not clearly specified then individuals may struggle to use them to review their own practice and identify continuing professional development opportunities to address their deficits. Given these standards were modelled on documents from other countries it is possible that they may have the same issue. As this was the first study of its kind to evaluate practice standards and more specifically pharmacist professional practice standards, we do not know to what extent this may be an issue outside the Australian context.

Behavioural specificity should be considered when writing practice standards for any profession, not just pharmacists. The researchers suggest that standard developers apply behavioural specification at the smallest possible unit or wherever an action is described. Specifying Actor, Context, Target and Time in other sections, such as the background or scope sections, may dilute important information and make it less accessible. Where behavioural information is separated out to background sections for formatting it should be sign posted as to which actions the information applies, for example ‘this standard applies to all community pharmacists regardless of current practice.’ The researchers acknowledge that the level of detail provided and length of the document would need to be balanced with practical useability for its intended audience. However, digital formats could serve to improve and tailor the user experience in this case (e.g. broad statements that when clicked on offered specified examples of the behaviour).

This study builds upon previous work to demonstrate that researchers can adapt behavioural specification frameworks to evaluate behavioural descriptions in guidelines, protocols and now for the first-time, professional practice standards [16, 18]. It confirms that this type of review can highlight areas of ambiguity that may prevent ease of interpretation and therefore implementation and evaluation of behaviours in these documents. Thus, with some adaptation these findings have highlighted the AACTT framework’s useability, applicability and acceptability in a setting where behaviour change and intervention design are not necessarily the immediate focus. The changes made to the framework in this study may serve as a consideration for future iterations of the framework to improve its applicability and use in broader settings. The processes undertaken are likely useful for other professional bodies and standard writers to consider when writing or reviewing their own practice standards, or even guidelines. The researchers suggest that when applying this approach to other documents for use in other contexts that research teams ensure representation from relevant disciplines to aid interpretation and application of the framework.

### Strengths and limitations

To the researchers’ knowledge, this is the first study to apply the AACTT framework to evaluate behavioural descriptions in practice standards. As this was not the original purpose of the framework, significant interpretation and adaptation by the research team was required. However, we ensured rigor in the process through the completion of extensive reliability checks throughout the process of developing the codebook and applying it across the dataset. This included discussion until consensus was reached on the sections of the standards reviewed and the definitions in the codebook, after multiple rounds of duplicate pilot coding on sections of the document. After consensus on the codebook was reached and it was applied to all included data, a final 20% second researcher check on the data was performed. The same two researchers conducted this entire process. One researcher had a professional background as a pharmacist and the second as a behavioural scientist, ensuring both relevant areas of expertise were represented in each reliability check. The results from this study are based on the evaluation of one practice standard, limiting their generalisability. It remains unclear if the lack of specificity is particular to the specific standards evaluated. However, it was deemed necessary to limit the study to one standard, in the first instance, to ensure that adequate detail in the process undertaken could be reported and replicated in future research.

Future work should investigate the extent to which these findings apply to other pharmacist practice standards internationally. Additionally, other health professions may benefit from reviewing their own practice standards and guidelines in this manner. It would also be beneficial to explore end user perceptions of barriers and enablers to using these documents as intended. Once appropriately specified, there are a plethora of tools to support understanding influences on behaviours and matching this to congruent intervention components (e.g. the Behaviour Change Wheel) [20].

## Conclusions

The Professional Practice Standards for Australian Pharmacists do not specify behaviours adequately which risks misinterpretation and a lack of consistency in professional practice. The lack of specificity also limits opportunities to observe and measure the behaviours of the profession and to develop interventions to increase the likelihood that desired professional behaviours are enacted. Behavioural specification frameworks, such as the AACTT framework, can be applied to shed light on the intrinsic characteristics of how specifically behaviours in practice standards are expressed. These results could be used to improve practice standards and guidelines across professions and disciplines in the future.

## Data Availability

The datasets used and/or analysed during the current study available from the corresponding author on reasonable request.

## Appendix

Table 7

**Table 7:** Consolidated criteria for reporting qualitative studies (COREQ): 32-item checklist

No. Item	Guide questions/description .	Page number reported on
Domain 1: Research team and reflexivity		
Personal Characteristics		
1. Interviewer/facilitator	Which author/s conducted the interview or focus group?	N/A Document review – the researchers background are described in the methods section on page 6
2. Credentials	What were the researcher’s credentials? E.g. PhD, MD	N/A Document review
3. Occupation	What was their occupation at the time of the study?	N/A Document review
4. Gender	Was the researcher male or female?	N/A Document review
5. Experience and training	What experience or training did the researcher have?	N/A Document review
Relationship with participants		
6. Relationship established	Was a relationship established prior to study commencement?	N/A Document review
7. Participant knowledge of the interviewer	What did the participants know about the researcher? e.g. personal goals, reasons for doing the research	N/A Document review
8. Interviewer characteristics	What characteristics were reported about the interviewer/facilitator? e.g. Bias, assumptions, reasons and interests in the research topic	N/A Document review
Domain 2: study design		
Theoretical framework		
9. Methodological orientation and Theory	What methodological orientation was stated to underpin the study? e.g. grounded theory, discourse analysis, ethnography, phenomenology, content analysis	Directed content analysis – methods sections, page 6
Participant selection		
10. Sampling	How were participants selected? e.g. purposive, convenience, consecutive, snowball	N/A Document Review. Rationale for the choice of document analysis can be found in the methods section, page 6
11. Method of approach	How were participants approached? e.g. face-to-face, telephone, mail, email	N/A Document Review.
12. Sample size	How many participants were in the study?	N/A Document Review.
13. Non-participation	How many people refused to participate or dropped out? Reasons?	N/A Document Review.
Setting		
14. Setting of data collection	Where was the data collected? e.g. home, clinic, workplace	N/A Document Review.
15. Presence of non-participants	Was anyone else present besides the participants and researchers?	N/A Document Review.
16. Description of sample	What are the important characteristics of the sample? e.g. demographic data, date	N/A Document Review.
Data collection		
17. Interview guide	Were questions, prompts, guides provided by the authors? Was it pilot tested?	N/A Document Review.
18. Repeat interviews	Were repeat interviews carried out? If yes, how many?	N/A Document Review.
19. Audio/visual recording		
20. Field notes	Were field notes made during and/or after the interview or focus group?	N/A Document Review. The researchers documented decisions during development of the code book.
21. Duration	What was the duration of the interviews or focus group?	N/A Document Review.
22. Data saturation	Was data saturation discussed?	N/A Document Review.
23. Transcripts returned	Were transcripts returned to participants for comment and/or correction?	N/A Document Review.
Domain 3: analysis and findings		
Data analysis		
24. Number of data coders	How many data coders coded the data?	Second researcher reviewed 20% of coding for agreement, see methods page 6–7
25. Description of the coding tree	Did authors provide a description of the coding tree?	Development of code book provided in methods, page 6–7 Code book can be viewed in Table 1
26. Derivation of themes	Were themes identified in advance or derived from the data?	Directed content analysis – methods sections, page 6
27. Software	What software, if applicable, was used to manage the data?	Microsoft Excel was used to store data and analysis.
28. Participant checking	Did participants provide feedback on the findings?	N/A Document Review
Reporting		
29. Quotations presented	Were participant quotations presented to illustrate the themes / findings? Was each quotation identified? e.g. participant number	N/A Document Review. Examples of analysis and potential improvements to statements can be viewed in Table 6
30. Data and findings consistent	Was there consistency between the data presented and the findings?	Yes.
31. Clarity of major themes	Were major themes clearly presented in the findings?	Yes. See results section on page 7–9
32. Clarity of minor themes	Is there a description of diverse cases or discussion of minor themes?	N/A Document Review

## Abbreviations

AACTT: Actor, Action, Context, Target, Time
TACT-A: Time, Action, Context, Target-Actor
PSA: Pharmaceutical Society of Australia
